# Editorial

**Published:** 1916-03

**Authors:** 


### EDITORIAL

The Atlanta Journal-Record of Medicine has its Business Office at 23 West Alabama Street where all business communications should be sent.

Remittances are payable to The Journal-Record of Medicine.

Concerning Original Communications and Editorial matters, address Richard R. Daly, M. D., 708 Empire Life Building, Atlanta, Ga.

Reprints of Original Articles will be furnished at cost price. Requests for them had best be made on the Manuscript.

Upon request, we will present, postpaid, to each contributor of an original article, twenty copies of The Journal-Record of Medicine containing such article.

### TESTIMONY.

Can one believe his own eyes? To have raised this question at one time would have set up a heresy against common sense and the advocate would have won the scorn of his hearers if he did not escape something more tangible and harmful. The statement is still made by many men as the clinching fact of testimony as to events in doubt—"I was there and saw it with my own eyes." Are they to be believed?

That they are honest will be granted and that their intentions are good and unshaped by desire to further one side or the other will be admitted, yet adverse statements are often made by equally credible witnesses whose eye sight has not been damaged and whose animus has not been impugned so far as one knows. These men believe their own eyes and they put a queer emphasis upon the possessive adjective. To particularize their "own" yes at once postulates the value of other eyes and the conclusions drawn from such alien observation, and the unconscious credit thus given to others perceptions shows that somehow and sometimes the protesting witness whose own eyes are not to be questioned, knows that he has to rely upon other things than his eyes and other eyes than his own for the truth.

To a jury and to the mass of the people direct statements of facts as seen are convincing and to all these people the value of circumstantial evidence is of such status that they hesitate to allow convictions of importance to rest upon the indirect testimony. The man in the street will denounce decisions even when ordered by eminent jurists if the dependance has been upon circumstances and what has been shown to be mistaken statement has been set aside.

The trained observer knows that he can not believe his own eyes all the time. He knows it better and better the more he studies and the nearer he perfects his perceptive powers. The broad-minded man seeks eagerly for the opinions of other qualified men to confirm or modify his observations and then asks closer consultation in simultaneous study. The real consultation in the sick room is an illustration and the case with which the incomer will occasionally clear up a doubtful case shows that he has seen something the first man has overlooked and continued to overlook. It does not necessarily mean that he is the better man of the two, but it results from his being fresh to the case and free from preconcepts.

An interesting illustration of the fallibility of eye-sight, appears in the reports of the large astronomical observatories of the country as to the surface of the planet Mars. Here are men most carefully trained to analyze what they see, to photograph it for their leisure contemplation, to act with seeming perfect freedom of mind and then to announce what is there. It doesn't matter in the least whether there are canals on Mars or not. Nothing depends upon it; our interest in the planet would be just the same if the astronomers were to announce that the sphere were becoming cuboid for unascertained and probably internal reasons and dissensions. Yet these men differ among themselves and become acrimonious and bitter like some of our contemporaries because we do not agree with them as to editorial policy, and they say that there are no canals on Mars or that there are definite lines showing canals. In either case the clinching argument is seeing with their own eyes.

One would say that a justice of a high court is likely to be competent to determine whether what he is looking at is so or not. His legal training has been along lines to make him a careful critic of evidence. He holds his high position in virtue of this ability. He is a "judge". Yet he steps aside from the ermine and says that the man before him is sick. He adds that he has ordinary common sense and he can see for himself that despite the testimony of medical men who have known the malingerer for three years, the man at the bar is sick now and entitled to the mercy of the court whose judgment he has succeeded in evading until this time. The man gets the benefit and grins into his shabby sleeve sympathetically but whether for himself of the misled jurist, he is careful not to say. One wonders what would have happened in the same court if a physician should have said that in the complicated case that preceded this, he knew what was common sense and despite the presence of the best legal men in the community, a matter of equity and ordinary fairness should prevail over decisions where statutes were involved and equity had no place. This striving to be expert in another man's field causes a lot of trouble which is but another way of saying that a man should mind his own business if he knows how. The men who know how for two are like the lion and the lamb as they lie down together in serenity—a sort of half-baked fable.

It is a common experience for a bank to get out of balance and stay out until the device of checking accounts or exchanging books is resorted to when each man has new columns to criticise. Then the error is seen which the perpetrator persistently missed because he had a habit of thought and made the same mistake every time he went over the column. He had every reason to make the correction; there was no animus leading him astray, but a quickly formed preconcept stood almost imperatively in the way of his percepts and he did not see with his own eyes correctly, or else did not know what he was looking at.

In the test types used in fitting glasses, there is sometimes inserted a square between two letters. If the patient sees this correctly and promptly, he certainly has been pretty well fitted for that line at least. He has overcome a handicap adroitly placed to confuse him and to add a special test of his visual acuity. It is surprising how many intelligent persons will insist upon calling the square an H or a U or an N when their very hesitancy tells the observer that they know it is not the letter called. They will persist in nominating some letter even when the physician asks with emphasis what *cha a er* is between the other letters. They cannot believe their own eyes because of the context; the presence of the contiguous letters suggests another letter so forcibly that the belief in their own eyes is overcome even when there is no excitement to confuse them or special interest to mislead them.

Thus when the question is asked—Can one believe his own eyes? The answer must be qualified in a large number of cases by the determination of the presence in the individual of some or all of the basal factors:

The profound tendency of men to leave their own field and attempt to shown wisdom in that of another.

Habit of thought as to stubbornness in decision or amenability to constructive criticism.

The presence of preconcepts as against openness of mind.

The affect on context.

One might say that in relating what he saw occur during a brief and unexpected event, these were all absent, but he misleads himself.

To take them in reverse order, there are always things happening before and after any event and no one knows how they color the picture sudenly thrown upon the screen of consciousness.

There is always in a man's mind some idea to come into relation with what he sees and this idea may be so well established as to be a preconcept without his instantly realizing it; it enters into the idea of the unexpected sight with the rapid flash of a dream and becomes incorporated with it in a dismaying fashion when his testimony is given in conflict with other evidence.

Habit of thought misleads the victim more than it does others but it is always in the way of correct interpretation when it is stubborn and of assistance when it is carefully analytic.

The tendency to do other's work and to see and announce things that will make such work better rests upon the superficial desire of many men to appear wise and it betrays their secret waking dreams that they are the unfortunate victims of adverse conditions which have kept them from being the shining lights in the world their abilities, if recognized, would make them. We all have it more or less and so it is comprehensible and viewed charitably, but it is sometimes awfuly in the way when it relates to testimony as to life and death. Sometimes it seems to be a temporary obsession. It is honest once it is started.

Thus if a man have one of these factors he must regulate it before he should ask anyone to believe his own eyes and if he have them all he had far better be blind. Seeing, he is a stubborn nuisance.

The process of changing and reforming ideas by memory after one has seen an event combines with the retelling of it to make the story diverge from the truth, but that is another matter as Kipling says. Can one believe his own eyes? Not implicitly even when they are shut.

---

### BERNARD WOLFF, M. D.

Dr. Bernard Wolff, a leading Dermatologist of Atlanta and the South, died March 14, 1916, following an operation for the diagnosis and relief of obscure symptoms. Exposure of the right kidney revealed a malignant condition that had already infiltrated the adjacent tissues, a continuation of which was inevitable—with its weeks of suffering. His death so early after operation was a merciful ending, the only compensation we have.

Dr. Wolff graduated in literary college, in medical college and then went to New York, where he took special training in various branches of practice. Deciding to take up Dermatology he obtained preliminary training there and then went abroad, spending the greater part of his time as a resident pupil with Unna of Hamburg. Later he went to Paris and London. He located in Atlanta in 1893, and soon established a substantial practice in his specialty.

He was lecturer on Dermatology in the Southern Medical College until this school and the old Atlanta Medical College consolidated into the College of Physicians and Surgeons in which college he continued his lectureship and later became clinical professor.

When this college and the Atlanta School of Medicine became the Atlanta Medical College and later the Medical Department of Emory University his professorship continued. For several years he was editor of the Southern Medical Record, going over as editor of the Atlanta Journal-Record of Medicine when the Southern and the Atlanta Medical and Surgical Journal united. His wonderful vocabulary and brilliancy of thought and expression rendered him uniquely effective as a teacher and writer. At the time of his death he was supervising editor of the Journal-Record of Medicine. He had been chairman of the City Board of Health, president of the Fulton County Medical Society and was the founder and president of the Atlanta G. U. and Derm. Society, a Mason of high degree, an ex-official.

Among his friends he had no title. He was just "Wolff"—distinct and distinguished. Destiny probably looks upon this earth as a shriveled planet, its people microbes good or bad. For each, perhaps, a task is set, those who do their work best and quickest being the first called away, the books closing. Others hang on through a long life and have little to show to their credit at the end. Passing just at 48, Wolff's life was short but in work accomplished and in gentleness disseminated the sum total could not be balanced by the calendar.

Scientific or not, there is a common belief that white contains all the colors, the combination obscuring those that are disagreeable, bringing the whole to a pleasing harmony.

Wolff was a mixture, with the "yellow" left out. He was a composite of human characteristics with the gall and acid omitted, a chemical end product of contradictions blended into attractive form. Stated as an equation it would be "Wolff—Brilliant mind, Amiable disposition, Personal magnetism."

In his social and busines relations he had the mind of a child—taking your candy or toys with innocent nonchalence, and, seemingly, just as well pleased if you took his. In answer to an angry letter about his neglect of editorial duties he said "I do not like to be chided like a child. The weather is hot and the mosquitoes bother me so I can not write. And, then, at the last moment he would overwhelm the printers with a basketful of copy.

His violations of conventions occurred in this same child-like spirit. Yet, in his practice and teaching he was reserved, dignified and firm. His friends were such without volition, enmity against him could not survive contact with or communication from him. The polarity of his magnetism would have drawn a practice had he been wanting in learning and intelligency, but he was an able and progressive physician.

His friendships and clientele embraced every class, from Jew to Gentile, financier to pauper, intellectual to imbecile.

Wolff's easy and amiable disposition was the more striking from the fact that his youth and early manhood were clouded with the dread of "hereditary" tuberculosis, the malady which carried off his father while still a young man. He further had to fight against a peculiar neurasthemia; little ailments, various fears and imaginings, but he "threw them off." This last illness began its mysterious and malignant creeping nearly two years ago. If he ever suspected the condition he kept his thoughts to himself. Having always a distaste for surgery, he was particularly reluctant to permit an exploratory operation until fate had got the cards stacked against him.

We like to believe that the balance in the Records is in his favor and that the gentleness, kindliness and charity in his life more than overweighed his inadvertant and child-like infractions of bromidic regulations. M. B. H.

### BERNARD WOLFF, AN APPRECIATION

Bernard Wolff, born March 27, 1868, in Prince Edward County, Virginia. He was of distingushed ancestry, his grandfather being James McDowell, governor of Virginia. His Revolutionary Grandfather was General William Campbell, who commanded the forces at the battle of King's Mountain. Patrick Henry was a great great uncle· He attended Hampden College Virginia, 1882-1886. He attended University of Virginia 1886-8, obtaining his M. D. 1888 at the age of 20 years. His distinction in his class won him the place as assistant demonstrator of anatomy, which place he filled at the University in 1888-89; he was a member of the Phi Kappa Si P. K. V. fraternity, also of the Eli Banana, a most exclusive social secret society of the University. He attended the P. & S. of Columbia University 1889-90, after which he served as resident physician in a number of the New York Hospitals, among them the Willard Parker, the Hospital for Contagious Diseases, the Chambers Street Emergency, etc. He went to Heidelberg, Germany in 1891 to take up the study of the specialty to which he devoted the remainder of his life and was a pupil of Dr. Green of Hamburg and a resident physician in Dr. Uma's sanatarium which had been moved to Heidelberg during the epidemic of cholera in Hamburg.

He settled in Atlanta on return from abroad in 1893 to practice his profession. In 1894 he married Marian Hillyer, the daughter of Judge George Hillyer and to them were born three children: Elmer McDowell, a student at Columbia College; Marian Hillyer and Bernard Preston. In 1895 his literary tendencies led him to undertake the editorship of the Southern Medical Record, afterwards known as the Atlanta Journal Record of Medicine· In collaboration with professional friends he continued to edit this magazine until a few years ago. His editorials being ever distinguished for their originality, brilliance and fluency. Not infrequently his contributions were in verse which he was often told it was superfluous to sign his name, as their authorship was unmistakable.

He was president of the Atlanta Medical Society, president of the Atlanta Board of Health, member for 1904 from the 5th district of the State Medical Commission on Tuberculosis, serving this as secretary. He was a professor at the Atlanta College of Physicians and Surgeons and had been elected to a professorship at Emory. Was a member of visiting staff at Grady Hospital and gave of his service to all the charitable institutions of the city. Member of Capital City Club and University Club. His Masonic affiliations were very dear to him. Was a member of Palestine Lodge F. & A. M. of which he was worshipful master in 1909. Member of Mt. Zion Chapter of Royal Arch Masons, of which he was High Priest in 1907-8. Member of Coeur de Lion Commandery of Knights Templars and of Yaarab Temple of Shrine.

Dr. Wolff was the author of Wolff's Practical Dermatology and was a constant contributor to the best known medical magazines over the country. His literary efforts were not confined to medical and technical subjects, as he had published a number of short stories and poems. Scattered among the papers in his desk were found many exquisite bits of verse, and unfinished writings. His literary pursuits had begun to claim more and more of his time. He was an omnivorous reader and few men of his time were more widely and diversely informed. None came in contact with him who did not feel the magnetism of his personality or the biliancy of his attainments and originality of his conversation.

